# Developing Cultural Self-Awareness for Intervention in Diversity Contexts: A Scoping Review

**DOI:** 10.1177/13634615261471969

**Published:** 2026-08-13

**Authors:** Nolwenn Gonzalez, Maeva Traverso, Yvan Leanza

**Affiliations:** 1 École de Psychologie, Université Laval, Canada

**Keywords:** cultural self-awareness, intercultural competences, cross-cultural competences, intervention, counseling, human sciences

## Abstract

In a diversity intervention context, the practitioner may have biases and stereotypes related to their own cultural background that will influence their approach. Developing cultural self-awareness could help reduce the manifestation of these biases and stereotypes. However, it is likely to be one of the least researched aspects of intercultural competence. This scoping study was conducted to provide an overview of how cultural self-awareness is addressed across disciplines related to interventions in diverse contexts. The methodology used followed that of the Joanna Briggs Institute. The different steps of the scoping review are the following: keyword selection and translation in the different selected databases; a first sorting of the selected studies according to the defined criteria, based only on the title and the abstract; a second sorting by reading the entire study; data extraction and finally, analysis of the results. In total, 25 studies were included. The results showed that it is indeed possible to develop cultural self-awareness through workshops that promote interaction among participants, whether they are students or professionals already in the workforce. However, this focus of the research is relatively new and underexplored in the literature.

White et al. (2018) emphasized that biases affect how people treat each other. Institutional agents, like health care professionals or educators, are not immune to these biases affecting their interactions with the institutions’ users. To address these risks, several researchers have developed an interest in intercultural competences.

It seems necessary to clarify here where we stand in the debate on the nature of competencies. Two main assumptions emerge from the literature. On the one hand, competence involves achieving and measuring a specific goal, as, for example, asserted by Hook et al. (2017). Competence is therefore defined, in a static way, as the knowledge, aptitudes, and behaviors applied in specific situations (Zotzmann, 2015). In this perspective, once a competency has been acquired, there is no need to work on or improve it.

On the other hand, Reynolds (2014) suggests that we can acquire and develop skills throughout our lives. This is a dynamic phenomenon that exists on a continuum and only ends at death. Several theoretical models of intercultural competences are based on this assumption, including those of Deardorff (2006) and Ting-Toomey and Kurogi (1998). Deardorff’s (2006) model, for example, is circular, presenting no end. In line with these dynamic models, this article will consider intercultural competencies as dynamic, as a lifelong ongoing process (Reynolds, 2014). In addition, the plural form is used throughout this article, as the authors of dynamic models usually consider intercultural competences to consist of a set of different competences or dimensions.

The models cited on intercultural competences aim to highlight the various factors necessary for effective intervention in diversity contexts. Perhaps one of the most widely used models in psychology and counseling is the one proposed by Sue and colleagues (1982), which organizes intercultural competences into three dimensions: “Counselor Awareness of Own Cultural Values and Biases,” “Counselor Awareness of Client's Worldview,” and “appropriate intervention strategies” (Ponterotto et al., 1995, pp. 640–643). Following Sue's work, other authors have developed models of intercultural competences. A 2009 literature review identified 22 such models (Bartel-Radic, 2009). In all of these models, cultural self-awareness and questioning one's own cultural viewpoint were recurring dimensions described as “cultural self-awareness” (Black & Wells, 2007; Sue et al., 1982). This phenomenon was defined by Sue and his colleagues (1982) as an understanding of how culture affects oneself. There is currently a consensus that cultural self-awareness is essential for intervention in a diversity context. For Fei (2015), cultural self-awareness would allow for dialogue and exchange between two individuals regardless of the context (Fei, 2015, p. 55). In a care context, Kraemer (1974) explained that it could enable professionals to identify what in their interpretation was causing them to make incorrect assumptions about the person in front of them, which could lead to diagnostic errors (Kraemer, 1974). This leads to questions about one's own cultural roots and attitudes and how these affect the therapeutic relationship and the bond of trust with the patient (Parker et al., 1998; Roysircar, 2004). Despite all these arguments in favor of the development of cultural self-awareness, it seems to have been understudied. For example, there has been no review of the literature specifically devoted to cultural self-awareness. Furthermore, there is no synthesis of the methods used by researchers to measure or observe this awareness and by trainers to develop it. This study aims to fill these gaps.

## Research Aims and Questions

The primary objective of this project was to conduct a literature review on the integration of cultural self-awareness as a specific training goal of intercultural training programs aimed at developing intercultural competences among health, social service, and education professionals. The secondary objectives were: (1) to catalog the pedagogical and research methods available for observing and developing this awareness and (2) to evaluate the impact on participants of the training using these methods. This study aimed at answering the following questions:

1. What are the characteristics of studies dealing with cultural self-awareness (population, year of publication, etc.)?
2. What pedagogical methods were used to develop self-cultural awareness?
3. What research methods were used to observe/measure self-cultural awareness?
4. What observed or measured effects resulted from this development?

## Method

The chosen method was a scoping review following the Joanna Briggs Institute (JBI) framework (Peters et al., 2022). As the study did not involve human subjects, it therefore did not require ethical approval. According to this approach, a scoping review consists of several stages. The main ones are: keyword selection according to the research aims, and translation in the different selected databases by making Boolean queries; a first sorting of the selected studies according to the defined criteria, based only on the title and the abstract; a second sorting by reading the entire study; data extraction and finally, analysis of the results.

For the record, the first author is a young white female psychology student and doctoral candidate. The second author is a young white female studying for a bachelor's degree in psychology. The third author is a white man, university professor of psychology with a PhD in Education. Due to our positionality, some details may have escaped us. However, inter-rater validation was used during different steps of the study and may have reduced these biases. Besides, the three authors of this article are migrants, and one is an expert in intercultural intervention. This may have contributed to greater sensitivity to certain topics, such as addressing prejudice or bias in articles. In addition, the studies present factual data which might limits the influence of bias.

### Inclusion and Exclusion Criteria

The inclusion criteria were as follows: studies written in French or English, studies evaluating the effect of training in intercultural competences with an explicit mention of cultural self-awareness in the training or in the results, and participants who were professionals in health, social services, and education. Studies written in languages other than French or English and studies without empirical data (theoretical papers, editorials, etc.) were excluded.

### Selected Databases

Based on a librarian's recommendations, 12 databases were queried: PsycInfo, ERIC, Social Work Abstracts, Medline, Social Services Abstracts, IBSS, Sociological Abstracts, Dissertation and Theses, Education Source, Anthropology Plus, and Web of Science (https://www.webofknowledge.com/). Additional searches were conducted in the French databases Pascal and Francis, Érudit, and Cairn.info. References of selected studies were reviewed to determine their inclusion in the scoping review.

### Data Extraction

After querying all databases, the studies were imported into Covidence software for two stages of screening. First, duplicates were removed and then screening was performed by two independent reviewers to achieve inter-rater agreement. In cases of disagreement about whether a study should be included, the first two authors discussed the matter. If consensus could not be reached, the third author was consulted. A total of 962 studies were initially identified in all databases after duplicate removal, and 25 were finally selected for data extraction performed by two independent reviewers. The different steps of the study selection are shown in the PRISMA flow chart (Figure 1).

**Figure 1.**
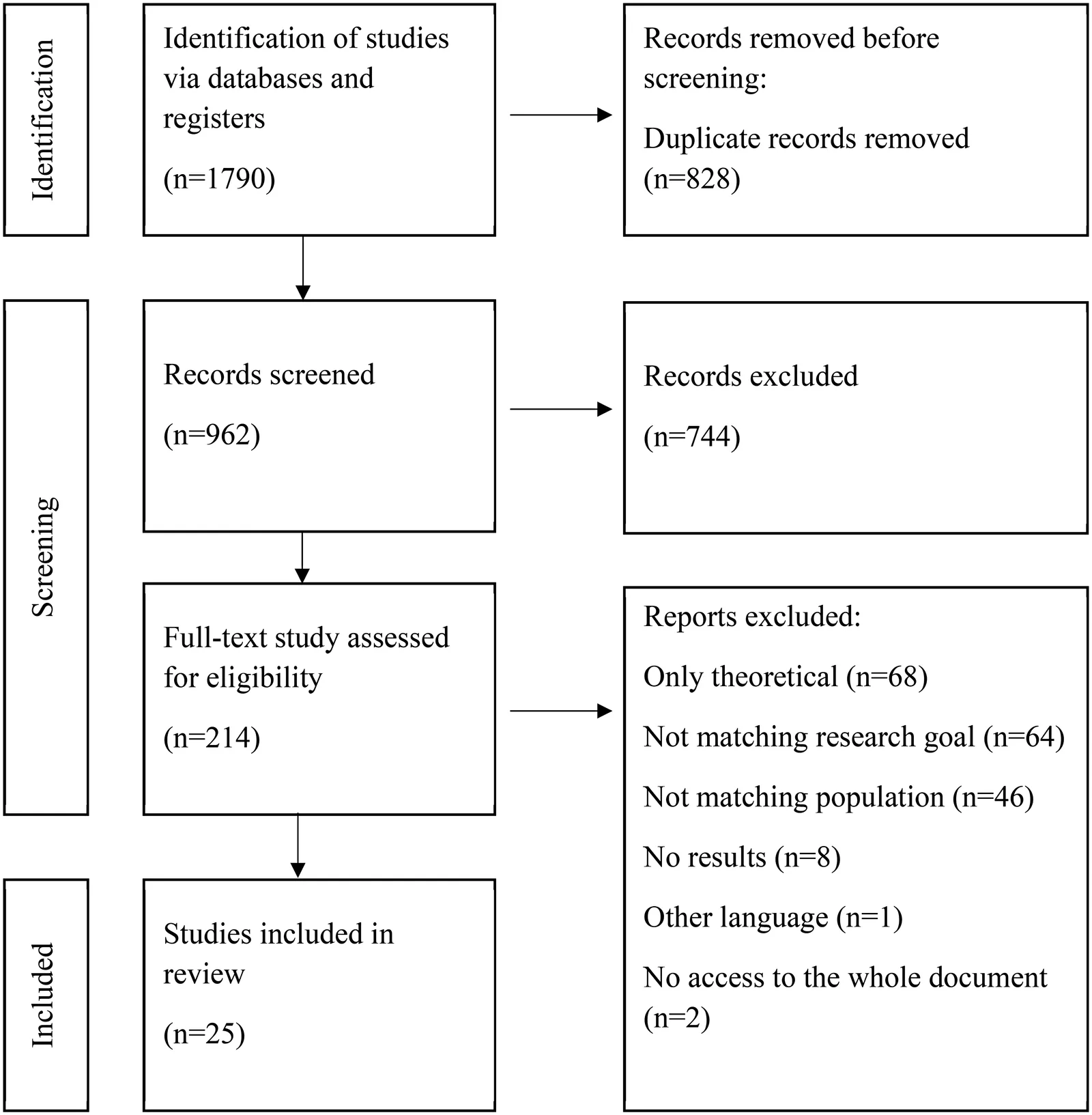
PRISMA study selection diagram.

Descriptive and content analyses were conducted to address the four research questions. The results were extracted using Excel web software. The content analysis involved a careful reading of the texts to identify recurring themes (Bardin, 2013). The information was organized into categories (pedagogical method, tools to measure cultural self-awareness, effects of training) and sub-categories (lecture, reflexion exercices, immersion; qualitative tools and quantitative tools). With regard to data validation, an inter-rater process was carried out during the categorization to increase its reliability. Analysis was then discussed between the first and last co-authors, the latter one being an expert on intercultural intervention.

Finally, a synthesis of the results drew meaningful conclusions in line with the research objectives (Zhang & Wildemuth, 2009). Table A1 (in the Appendix) presents the main characteristics of the 25 selected studies.

## Results

Results are presented as follows: description of the studies, pedagogical and research methods, and effects of the training programs.

### Descriptions of Included Studies

The search results showed that 92% (n = 23) of the studies were published after the 2000s (see Figure 2). All of them were in English, as no results meeting the inclusion criteria were found in the French databases. In addition, most of the included studies were conducted in the United States (84%, n = 21), while others were from the United Kingdom (4%, n = 1), Israel (4%, n = 1), Canada (4%, n = 1), and Australia (4%, n = 1). Nine studies did not report any sociodemographic characteristics of their participants, two studies reported the gender of participants but did not provide any other sociodemographic details, and one study reported only the origin of participants without specifying their gender (refer to Table A1). When these data were presented, it appeared that most participants were female (75.4%) and white (64.6%). These results were found by extracting the socio-demographic characteristics of the samples in each study. There were also participants from other backgrounds: African American (4.6%), Asian American (10.4%), Latino or Hispanic (5.2%), biracial or multiracial (2%), and other (13.2%). The “other” category included all non-Whites in two studies (Sawrikar, 2020; Wesselkamper, 1983). In addition, 88% of the studies (n = 22) were based on student populations, while 12% involved practicing professionals (n = 3).

**Figure 2.**
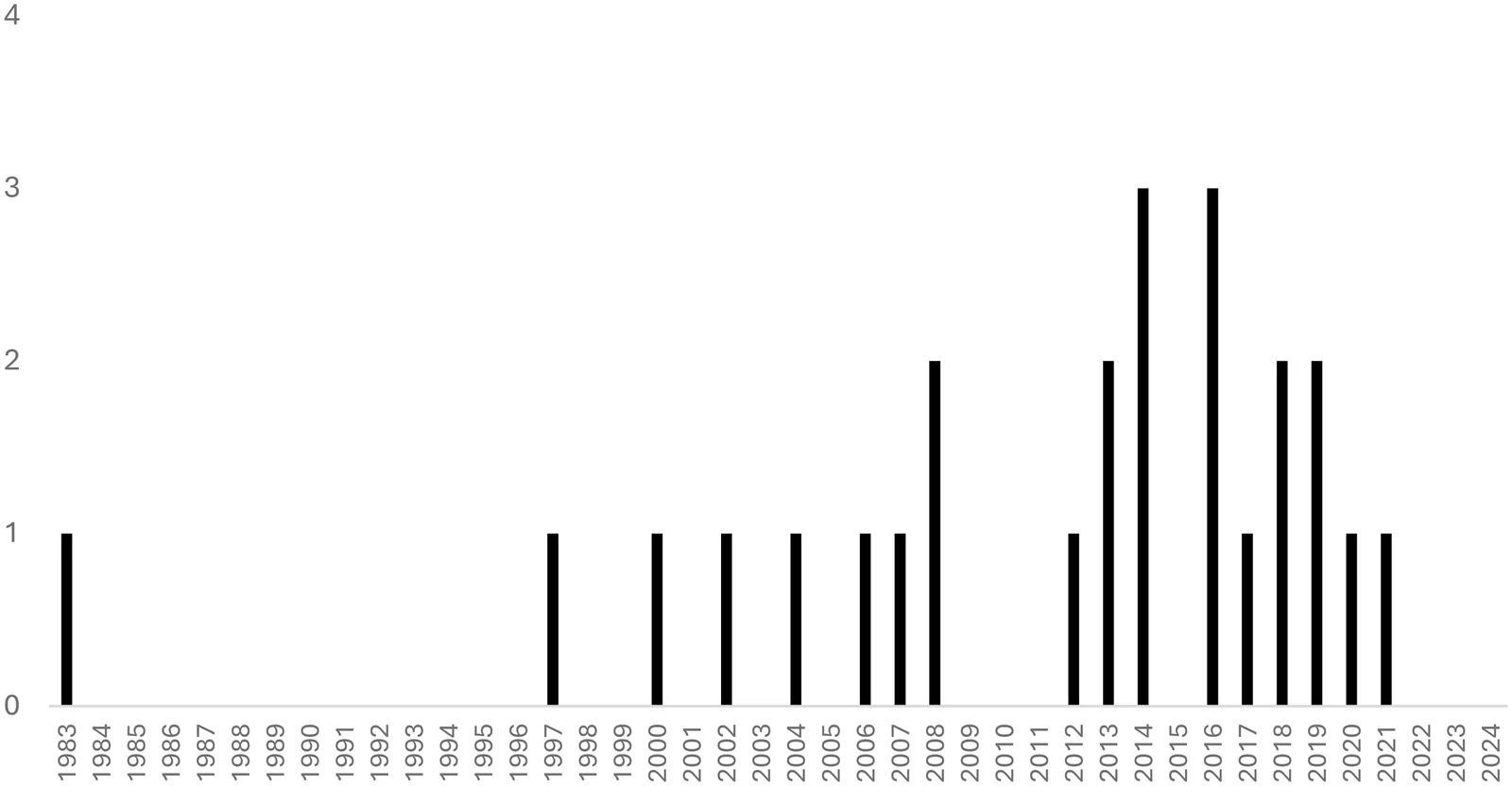
Publication dates of the studies.

**Table 1.** Pedagogical Method.

Method Type	Method Description
Lectures / interactive class	Theoretical lectures given to participants with an interactive approach where participants discussed with each other
Reflection exercises	Exercises given to participants to encourage reflection on their cultural backgrounds, behaviors, and thoughts
Immersion	Participants were immersed in another culture for several weeks

Regarding ethical approvals for the various studies, 14 studies (56%) mention having obtained approval, while 11 studies (44%) do not explicitly mention it. Nine articles that did not mention ethical approval were published between 2000 and 2021 (Alraqiq et al., 2021; Castillo et al., 2007; Fuller et al., 2006; Matkins et al., 2014; Mirsky, 2013; Parcells & Baernholdt, 2014; Smolcic & Arends, 2017; Spears, 2004; Tomlinson-Clarke, 2000). There are also those of Wesselkamper (1983) and Culhane-Pera et al. (1997). The majority (n = 10) of the articles that did not mention an ethnical approval are from the United States (Alraqiq et al., 2021; Castillo et al., 2007; Culhane-Pera et al., 1997; Fuller et al., 2006; Matkins et al., 2014; Parcells & Baernholdt, 2014; Smolcic & Arends, 2017; Spears, 2004; Tomlinson-Clarke, 2000; Wesselkamper, 1983).

The included studies focused on the development of intercultural competences rather than specifically on cultural self-awareness. Cultural self-awareness was addressed in the studies either because it was measured in the questionnaires used (Callo, 2002; Castillo et al., 2007; Conway, 2008; Kutob et al., 2013; Richards & Doorenbos, 2016) or because it emerged in participants’ discourses (Conway, 2008; Culhane-Pera et al., 1997; Fuller et al., 2006; Havis, 2019; Hosokawa, 2012; Hutnik & Gregory, 2008; Matkins et al., 2014; Merrell-James et al., 2019; Mirsky, 2013; Parcells & Baernholdt, 2014; Prosek & Michel, 2016; Richards & Doorenbos, 2016; Rubio Rodriguez, 2015; Sawrikar, 2020; Smith-Augustine et al., 2014; Smolcic & Arends, 2017; Spears, 2004; Tomlinson-Clarke, 2000; Tormala et al., 2018; Wesselkamper, 1983; White et al., 2018). Only one study, by Alraqiq et al. (2021), focused exclusively on the development of cultural self-awareness, in which the researchers evaluated the effectiveness of an intervention on the development of cultural self-awareness in dental students.

The primary disciplines represented in the studies included medicine (24%, n = 6), counseling (20%, n = 5), social work (20%, n = 5), psychology (16%, n = 4), and education (cited as “educators” in the studies) (12%, n = 3). Other disciplines such as criminology, dentistry, family therapy, gerontology, psychiatry, and pharmacy were also represented. Some studies included multiple disciplines (16%, n = 4). For example, Hosokawa's (2012) study included participants from clinical psychology, social work, gerontology, and family and couples’ therapy.

Different theoretical models were used in the studies’ designs. Two models, Sue's (1982) (16%, n = 4) and Bennett's (1986) (16%, n = 4), together accounted for nearly a third of the studies. However, most did not clearly specify a theoretical model (56%, n = 14). Regarding data collection methods, 72% (n = 18) of the studies used qualitative methods, 12% (n = 3) used quantitative methods, and 16% (n = 4) used a mixed methods design.

Several methods were used to evaluate the effectiveness of training programs. The most common method used was to assess the development of participants’ thinking during the workshop or immersion (n = 10), followed by pre- and post-intervention measurements (n = 6), post-intervention-only measurements (n = 5), measurements combining pre- and post-intervention measurements and a control group (n = 3), and finally the use of a control group to compare with the training group (n = 1). However, there were subtleties. Two studies only measured post-intervention, using two measurement points, just after the intervention and four months after (Tomlinson-Clarke, 2000) or six months after (Sawrikar, 2020). There were also studies that made assessments during workshops or immersions and just after the return to the United States or just after the workshop (Smith-Augustine et al., 2014; Tormala et al., 2018) or three weeks after (Merrell-James et al., 2019). Finally, three studies examined actions taken throughout the process, using a critical incident journal (Richards & Doorenbos, 2016; Rubio Rodriguez, 2015; Smith-Augustine et al., 2014).

### Pedagogical Methods

The pedagogical methods used by researchers to develop intercultural competences and cultural self-awareness were grouped into three categories: (1) lectures where participants could also exchange ideas (32%, n = 8); (2) exercises where participants reflected on their cultural backgrounds (52%, n = 12); and (3) immersion in another culture (16%, n = 5). The pedagogical methods are described in Table 1.

**Table 2.** Measures of Cultural Self-Awareness in Quantitative or Mixed-Design Articles.

Author(s) (Date)	Measurement Tools	Psychometrics Qualities	Source Study
Callo (2002)	Interpersonal Intercultural Functioning Rating Scale (IIFRS) and Multicultural Awareness-Knowledge-Skill Survey (MAKASS-C) (English)	IIFRS: test-retest reliability: Empathy subscale (.38); Respect (.58), Immediacy (.59), Cultural Intentionality (.85), and Counseling Technique (.90). Inter-rater reliability: Empathy (r = .64, p < .01, two-tailed), Respect (r = .75, p < .01, two-tailed), Immediacy (r = .53, p < .01, two-tailed), Cultural Intentionality (r = .29), and Counseling Technique (r = .61, p < .01, two-tailed). MAKASS: internal coefficient alphas of .75 for the Awareness subscale, .90 for the Knowledge subscale, and .96 for the Skills subscale. No coefficient alpha was reported for the total scale.	Callo, E. A. V. (2002). *The impact of the experiential multicultural counseling training program on counselor trainee cross-cultural competence*. [Unpublished PhD thesis, University of San Francisco] D’Andrea, M., Daniels, J., & Heck, R. (1991). Evaluating the impact of multicultural counseling training. *Journal of Counseling & Development*, *70*(1), 143–150. https://doi.org/10.1002/j.1556-6676.1991.tb01576.x
Castillo et al. (2007)	Multicultural Counseling Inventory (MCI), and Race Implicit Association Test (Race-IAT) (English)	MCI: average overall reliability of .87 for the MCI with mean coefficient alphas of .78, .77, and .80 for the Awareness, Knowledge, and Skills subscales. Race-IAT: convergent validity of the Race IAT with other established latency-based implicit racial attitude measures as well as with physiological measures of racial preference has been found. Test–retest reliabilities of r = .65 and r = .69 have been reported.	Sodowsky, G. R., Taffe, R. C., Gutkin, T. B., & Wise, S. L. (1994). Development of the Multicultural Counseling Inventory: A self-report measure of multicultural counseling competencies. *Journal of Counseling Psychology*, 41, 137–148 Greenwald, A. G., McGhee, D. E., & Schwartz, J. L. K. (1998). Measuring differences in implicit cognition: The Implicit Association Test*Journal of Personality and Social Psychology*, 74, 1464–1480
Conway (2008)	Intercultural Development Inventory (IDI) (English)	Denial/Defense scale: alpha = 0.85; Reversal scale: alpha = 0.80; Minimization scale: alpha = 0.83; Acceptance/Adaptation scale: alpha = 0.84; Encapsulated Marginality scale alpha = 0.80.	Hammer, M. R., Bennett, M. J., & Wiseman, R. (2003). Measuring intercultural sensitivity: The intercultural development inventory. *International Journal of Intercultural Relations*, 27(4), 421–443. https://doi.org/10.1016/S0147-1767(03)00032-4
Kutob et al. (2013)	Cultural Competence Assessment Tool (CCAT) (English)	The modified CCAT showed good internal reliability, with Cronbach's alpha ranging from 0.77–0.93 (n = 90) on all subscales, 0.89–0.93 on the 3 modified subscales (n = 90), and an overall alpha of 0.94.	Kutob, R. M., Senf, J. H., & Harris, J. M., Jr (2009). Teaching culturally effective diabetes care: Results of a randomized controlled trial. *Family medicine*, *41*(3), 167–174
Richards & Doorenbos (2016)	Intercultural Effectiveness Scale (IES) and Intercultural Sensitivity Scale (ISS)	IES: The scale has acceptable reliability (α = .85) and has face and content validity. ISS: The ISS has acceptable reliability (α = .86), with good predictive validity and concurrent validity with five other related instruments.	Portalla, T., & Guo-Ming, C. (2010). The development and validation of the intercultural effectiveness scale. *Intercultural Communication Studies*, *19*(3), pp. 21–37. https://web.uri.edu/iaics/files/02TamraPortallaGuo-MingChen.pdf Chen, G. & Starosta, W. (2000) The development and validation of the intercultural sensitivity scale. *Human Communication*, 3, 1–15. https://doi.org/10.1037/t61546-000
Sawrikar (2020)	General satisfaction questionnaire with specific questions on cultural self-awareness		“How self-aware of your own cultural background do you feel you are?” “Have you ever heard of the phrase ‘White privilege’?”

#### Lectures / Interactive Classes

Conventional methods such as theoretical or lecture-based courses were used. Sometimes participants were also shown videos and had to read papers (Castillo et al., 2007; Conway, 2008; Culhane-Pera et al., 1997; Sawrikar, 2020; Spears, 2004; White et al., 2018). Common to all these studies was the fact that while the content was primarily theoretical, instructors also encouraged interaction with participants to enhance reflection on intercultural issues (Parcells & Baernholdt, 2014; White et al., 2018). Among the various studies reviewed, researchers proposed participants consider a different approach to think about their cultural background. They were encouraged to read studies, watch documentaries, or do exercises like the wheelchair exercise where participants imagined spending a day in a wheelchair to appreciate their privileges. Afterwards they talked about their feelings in a group discussion (White et al., 2018). There were also group discussions about what intercultural competence entails and the influence of cultural values (Parcells & Baernholdt, 2014).

#### Reflective Exercises

Most studies (44%, n = 11) included reflective exercises given to participants. A variety of these exercises had the primary aim of developing intercultural competences, but also contributed to the development of cultural self-awareness. The themes addressed were identity (Alraqiq et al., 2021; White et al., 2018), power (Alraqiq et al., 2021; White et al., 2018), privilege (Alraqiq et al., 2021; Smolcic & Arends, 2017; White et al., 2018), communication (Alraqiq et al., 2021), prejudice (Fuller et al., 2006), bias and stereotyping (Mirsky, 2013; White et al., 2018), and self-reflection (Alraqiq et al., 2021). In all the studies that focused on reflection exercises, researchers also used discussion among participants to enhance reflection.

Among the exercises designed to stimulate introspection, specific methods included the genogram (Hutnik & Gregory, 2008; White et al., 2018), autobiography (Fuller et al., 2006), reflecting on the origin of one's first or last name (Hutnik & Gregory, 2008), and questioning the symbols of the participant's culture (Callo, 2002).

Another widely used type of exercise in the studies involved interviews with individuals from different cultural backgrounds (Fuller et al., 2006; Havis, 2019; Mirsky, 2013; Smolcic & Arends, 2017). The purpose of these interviews was not only to listen to a different life experience but also to question one's own background, reflect on what it means to interact with others, and address issues of microaggression (Fuller et al., 2006; Havis, 2019; Mirsky, 2013; Smolcic & Arends, 2017).

Regarding reflective exercises focused on clinical practice, one method involved the presentation of clinical vignettes. Participants were tasked with suggesting treatments that considered the cultural differences of the presented client (Kutob et al., 2013; Tormala et al., 2018). In Rubio Rodriguez's (2015) study, the use of a critical incident journal between consultations with refugees facilitated self-reflection and a consideration of factors influencing practice.

#### Immersion

Immersion in another culture was found in four studies (Merrell-James et al., 2019; Prosek & Michel, 2016; Richards & Doorenbos, 2016; Smith-Augustine et al., 2014). In three of these, participants underwent pre-immersion sessions that covered several topics: expectations (Merrell-James et al., 2019), exploration of intercultural issues (Merrell-James et al., 2019), exploration of one's own values and beliefs (Richards & Doorenbos, 2016), and reading and reflection on the host culture, its values, and history (Prosek & Michel, 2016).

Participants did not necessarily need to travel abroad to immerse themselves in another culture, as demonstrated by Matkins and colleagues (2014). The researchers compared the effectiveness of two immersion approaches among diverse populations. In the first approach, participants were immersed in a high-needs school setting. In the second approach, participants were immersed in a similar school setting but also engaged in exercises designed to stimulate reflection on cultural issues, including cultural self-awareness (Matkins et al., 2014).

Several immersion studies included discussions among participants to further stimulate reflection. Authors asked questions such as, “What did you learn about the culture you were in?” (Merrell-James et al., 2019) and “How did this experience affect you?” (Prosek & Michel, 2016). These discussion groups could take place during immersion (Merrell-James et al., 2019; Smith-Augustine et al., 2014) or after immersion (Merrell-James et al., 2019; Prosek & Michel, 2016).

### Pedagogical and Research Methods

Qualitative data were collected from reflective journals that also served as teaching tools (Richards & Doorenbos, 2016; Rubio Rodriguez, 2015; Smith-Augustine et al., 2014), from questionnaires with open-ended questions (Conway, 2008; Fuller et al., 2006; Hosokawa, 2012; Sawrikar, 2020; Spears, 2004; White et al., 2018), from individual interviews (Alraqiq et al., 2021; Culhane-Pera et al., 1997; Hutnik & Gregory, 2008; Matkins et al., 2014; Merrell-James et al., 2019; Tomlinson-Clarke, 2000), from focus groups designed to elicit questions and reflections from participants (Culhane-Pera et al., 1997; Merrell-James et al., 2019; Prosek & Michel, 2016; Smith-Augustine et al., 2014), and from reports submitted by participants at the end of the intervention (Havis, 2019; Mirsky, 2013; Smolcic & Arends, 2017). In terms of quantitative outcomes, the data collection instruments are presented in Table 2.

### Effects of Training

#### Lectures / Interactive Classes

*Studies using quantitative methods*. Castillo et al.'s (2007) study showed a significant difference between pre-test and post-test scores on the Cultural Self-Awareness subscale of the Multicultural Counseling Inventory (MCI), with higher scores observed after participants had received 15 classes of three hours on multicultural counseling (Castillo et al., 2007). The difference between the pre- and post-test cultural self-awareness scores was higher for the program group compared to the control group. A similar finding was found in Sawrikar's (2020) study with a satisfaction survey and a specific question which was: “How self-aware of your own cultural background do you feel you are?” The results showed a slight increase in participants’ cultural self-awareness scores from pre- to post-intervention; however, this difference was not statistically significant (Sawrikar, 2020).

*Studies using qualitative methods*. It is interesting to note that several studies reported some form of resistance by participants when asked to engage in self-reflection (Culhane-Pera et al., 1997; Tomlinson-Clarke, 2000). Despite this resistance, participants in Tomlinson-Clarke's (2000) study expressed that a course emphasizing cultural self-development would be highly beneficial in overcoming the biases and stereotypes of which they had become aware.

Concepts addressed by participants included White privilege and questioning their own interpretations of events (Sawrikar, 2020). In Spears’ (2004) study, participants reported an increased critical awareness of their own biases, which contributed to the development of cultural self-awareness (Spears, 2004).

#### Reflection Exercises

*Studies using quantitative methods*. Quantitative results from studies using reflection exercises did not show an effect of training on the development of cultural self-awareness. In Kutob et al.'s (2013) study with a 9 h course divided into modules, results indicated that the Cultural Competence Assessment Tool (CCAT) Cultural Self-awareness subscale score was lower for the intervention group (3.26) compared to the control group (3.48), with an effect size of 0.52 and *p* = .018. In Callo's (2002) study, there was no significant difference in cultural self-awareness between the control and intervention groups.

*Studies using qualitative methods*. In general, qualitative findings from these studies showed that participants reported an increase in their cultural self-awareness (Fuller et al., 2006; Hutnik & Gregory, 2008; Rubio Rodriguez, 2015; Tormala et al., 2018; Wesselkamper, 1983; White et al., 2018). Encouraging interaction and promoting dialogue among participants led to the emergence of specific themes during group discussions, including stereotypes, prejudices, ethnocentrism, and bias (Rubio Rodriguez, 2015; Tomlinson-Clarke, 2000; Wesselkamper, 1983; White et al., 2018). There was an awareness of one's biases and privileges, as seen in Fuller et al.'s (2006) findings, where one participant remarked: “The analysis helped me to be aware of the biases I have towards other cultures” (Fuller et al., 2006).

Several studies also found that participants perceived a less thorough understanding of their own culture than expected (Conway, 2008; Hosokawa, 2012; Rubio Rodriguez, 2015; Smolcic & Arends, 2017). This finding was consistent with the results of the study conducted by White and colleagues (2018), in which participants reported becoming aware of the existence of “blind spots”: “The most consistent and powerful theme was that participants felt the course opened their eyes to their personal biases and blind spots” (White et al., 2018, p. 6).

The idea that participants became aware of their own lack of knowledge set the stage for an expressed need in two studies: understanding oneself precedes understanding others and their respective cultures (Hutnik & Gregory, 2008; Parcells & Baernholdt, 2014).

The study by Fuller et al. (2006), which focused on the effects of cultural self-analysis exercises with 26 undergraduate pre-service educators, stood out for its practical approach to developing cultural self-awareness. In the study, participants engaged in various exercises such as autobiography, biography, intercultural analysis, cultural difference analysis, home connection, and reflection. Outcomes of the intercultural competences intervention included the development of tools and plans to increase participation and communication with parents from different cultures. However, the researchers did not examine whether these were put into practice by the participants (Fuller et al., 2006).

Wesselkamper's (1983) study, conducted with social work students in groups of various sizes (N = 8, N = 24, N = 7), is the only study to have highlighted a challenge associated with group discussions of intercultural competences. Discussions about ethnocentrism, stereotypes, and prejudice sometimes led to conflicts among participants (Wesselkamper, 1983). The study found that resolving these conflicts could lead to greater cultural self-awareness. However, when conflicts were not resolved, they did not contribute to changes in awareness (Wesselkamper, 1983).

#### Immersion

*Studies using quantitative methods*. Richards and Doorenbos (2016) used immersion to encourage participants to reflect on intercultural issues in health-related professions. However, scores on the Intercultural Effectiveness Scale (IES) and the Intercultural Sensitivity Scale (ISS) did not show significant differences between pre-test and post-test.

*Studies using qualitative methods*. Qualitative findings revealed participants’ awareness, often through deep introspection, as reported in Smolcic and Arends’ (2017) study. Participants first became aware that they were less sensitive to intercultural issues than they had previously thought (Smith-Augustine et al., 2014; Smolcic & Arends, 2017). They also recognized their lack of awareness of personal values and biases, and their difficulties in recognizing and suspending judgment, but they showed a willingness to change (Richards & Doorenbos, 2016). These realizations of insensitivity or bias sparked a desire in participants to learn more about their own cultural heritage (Smith-Augustine et al., 2014).

## Discussion

The discussion will be organized according to the research questions: overview of studies examining cultural self-awareness, pedagogical methods for developing cultural self-awareness, assessment of cultural self-awareness, and the impact of training on cultural self-awareness.

From a theoretical perspective, this literature review identified several aspects of cultural self-awareness: cultural values and representations (Richards & Doorenbos, 2016), prejudices (Wesselkamper, 1983), stereotypes (Parcells & Baernholdt, 2014; Smolcic & Arends, 2017), biases (Merrell-James et al., 2019; White et al., 2018), privilege (Alraqiq et al., 2021; Merrell-James et al., 2019), and power relations (Alraqiq et al., 2021). Cultural self-awareness appeared as a multidimensional construct. A more precise definition could then be: *the awareness of one's own prejudices, stereotypes, biases, privileges, and power relations in relation to one's cultural background(s)*. The first step toward cultural self-awareness is to become aware of one's own unconscious (Tomlinson-Clarke, 2000).

### Overview

The main results concerning the characteristics of the studies selected showed that they were mainly carried out with White women, after the 2000s, in the UK and in former UK colonies (United States, Australia …) and the main topic of the research was cultural and intercultural competences but not cultural self-awareness itself.

The fact that most study samples were made up of White women is representative of the helping relationship population (Kilminster et al., 2007; Odic &Wojcik, 2019). Nevertheless, it is debatable why these are predominantly female disciplines. One explanation might be that women are traditionally associated with the role of caregiver, taking care of family members, so it would be natural for them to turn to care-related disciplines (Nadot, 2014). This reflects the gender stereotypes that are ingrained in the society and that influence people's career orientation (Jaoul-Grammare, 2024). A study conducted by Gadassi and Gati (2009) on the direct and indirect occupational preferences of men and women showed that direct preferences showed a strong gender bias. Men chose “masculine” professions and women “feminine” professions. This bias was less present in indirect preferences, especially among women, showing that women are the most affected by gender stereotypes in their occupational choices. This underscores the need to deconstruct gender stereotypes and bias and to promote diversity in the workplace (Raj et al., 2023). The development of cultural self-awareness can be used to deconstruct those biases.

Furthermore, the predominance of White participants in the selected studies highlights a lack of diversity in the helping professions, which does not represent the diversity of the population (Villeneuve, 2002). This lack of diversity in research samples seems a general problem, at least in psychology: a study conducted in 2018 on samples from the studies published in the journal *Psychological Science* from 2014 to 2017 showed a systematic lack of diversity in the samples (Rad et al., 2018). In addition, most of the samples were from North America, without much information on the characteristics of the participants, which represents a strong limitation on representativeness and generalization (Rad et al., 2018).

According to statistics, in the United States, non-White people represent 34.12% of the population (Neilsberg, 2024). There is a disparity in the representation of visible minorities in nursing, as in other health professions (Villeneuve, 2002). This discrepancy highlights an inconsistency between the proportion of non-White people in the general population and their presence in the selected studies. Under-representation of non-White people in different professions can be explained by biases during recruitment, with a tendency to favor White candidates (Wingfield & Alston, 2014). In addition, people from diverse backgrounds may be reluctant to take part in research projects for fear of being discriminated against or of research abuse (Giuliano et al., 2000; Gross & Gelman, 2025; Schmotzer, 2012; Shea et al., 2022). This leads to an under-representation of people with diverse backgrounds not only in organizations but also in research samples. For future research on cultural self-awareness, it is imperative to acknowledge the necessity of diversity in the sample.

The modest number of studies and the predominance of those conducted after the year 2000 indicate that cultural self-awareness is still a relatively underexplored topic even though interest in the subject dates to the 1970s. It seems that the ever-increasing population and social movements like “Black Lives Matter” and “Idle No More” in recent years have led researchers to take a greater interest in intercultural issues, which could explain the increase in studies after 2000 (Pierce, 2017). Conversely, the preponderance of research concentrating on intercultural competences while neglecting or scarcely addressing cultural self-awareness may signify the inherent challenges in introspection, as this process can be distressing (Furlong & Wight, 2011).

The majority of these studies were conducted in the United States, Canada, the UK, or Australia and were subsequently published in English. This phenomenon can be attributed to the preponderance of theoretical models of intercultural competences originating from Western English-speaking countries (Bennett, 1986; Deardorff, 2012; Sue et al., 1982). Consequently, a unidirectional continuity of thought exists from theoretical to practical models, with the resultant interventions.

Regarding the disparities in ethical approvals among the selected articles, one possible explanation for studies that do not mention ethical approvals may be the date on which the study was conducted. Ethical approval requirements have changed significantly since 1983. For instance, the incident in the 1990s involving unauthorized use of human tissue for research in an English hospital led to a reconsideration of ethical approvals and an expansion of their scope to disciplines other than medicine (McAreavey & Muir, 2011).

Another assumption regarding ethical approval is that it is not always necessary when evaluating a course. In fact, according to the Tri-Council Policy Statement guidelines in Canada (2018), the analysis of anonymized secondary data, such as institutional course evaluations, does not constitute research involving human subjects and therefore does not require ethical approval. This position is consistent with that of other organizations such as the American Educational Research Association (2011) and the U.S. Department of Health and Human Services (2018). As a result, anonymized course evaluations are frequently used as pre-existing institutional data that does not require ethical approval.

### Pedagogical Methods

It is noteworthy that an important portion of the training programs lacked detailed content descriptions, and when provided, they were vague. In addition, cultural self-awareness was not always a central theme of the training programs, making it difficult to determine how it was addressed in trainers’ discussions and what specific activities were used to foster its development.

The results indicated a prevalence of training programs that emphasized reflective exercises and interaction among participants, with some using immersion. This is consistent with the recommendations of several authors to limit strictly lecture-based courses as they are less effective (Bezrukova et al., 2016; Bhawuk, 1990). Furthermore, as confirmed in a systematic review, immersion has positive effects from both a cognitive and an affective point of view, as well as improving cultural sensitivity (Brock et al., 2019). The results of this review also showed that immersion led to an increased awareness of how to interact with people from other cultures. As such, immersion as a pedagogical method could improve care provision in intercultural contexts.

### Assessment of Cultural Self-Awareness

Several studies included in the review used standardized questionnaires (Callo, 2002; Castillo et al., 2007; Conway, 2008; Kutob et al., 2013; Richards & Doorenbos, 2016). However, these questionnaires had limitations: they may not have been suitable for use with diverse populations, their psychometric properties were often unconvincing due to small sample sizes at the time of development, or their items may have lacked clarity (Matsumoto & Hwang, 2013). Most results obtained by quantitative methods did not seem to show an effect of training on the development of cultural self-awareness. There are a few explanations for these findings: (1) as discussed above, there is debate about the validity of the questionnaires used (Matsumoto & Hwang, 2013), or (2) participants may have overestimated their cultural self-awareness before the intervention and may have become aware of this overestimation afterward. This phenomenon is known as the Dunning-Kruger effect, in which individuals tend to overestimate their competences in areas in which they are not necessarily proficient (Kruger & Dunning, 1999). Kruger and Dunning also showed that the best way for people to recognize their incompetence is to make them competent (Kruger & Dunning, 1999), which is reflected in the results of studies in which participants rated themselves as more culturally competent before rather than after training sessions.

The use of qualitative methods partially addressed these limitations of quantitative instruments. Qualitative methods can capture the processes at play and provide a nuanced understanding of change (Leung et al., 2014). This dual perspective—the low reliability of questionnaires and the ability to capture nuanced change—may explain why qualitative methods were more commonly used in the reviewed studies. These qualitative methods included interviews with participants, open-ended questions, and diaries.

These contradictory results may also be because the construct itself is still poorly understood and its definition is sometimes confused with the concepts of cultural sensitivity and cultural humility (Lantz-Deaton & Golubeva, 2020; Tervalon & Murray-Garcia, 1998). Adopting a clear definition of cultural self-awareness, as the one proposed above, should lead to a better choice of instruments by which to measure it. To obtain reliable results, it would be worthwhile to revise the questionnaires used so that they have better psychometric qualities and to address the limitations often mentioned in the literature (Matsumoto & Hwang, 2013). It would also be worthwhile to develop a new definition of cultural self-awareness. Nevertheless, when it comes to evaluating cultural self-awareness, it seems qualitative data are still essential for effectively capturing the participants’ development processes. For this reason, studies of cultural self-awareness should systematically adopt a qualitative or mixed approach, and not a quantitative-only approach. Such studies appear very limited due to the shortcomings of existing questionnaires. The use of a mixed methods design provides valid quantitative data and complementary information through a deeper understanding of phenomena thanks to qualitative data (Daymon & Holloway, 2010).

### The Impact of Training on Cultural Self-Awareness

The different results from the studies reviewed addressed the final research question: What effects were observed or measured from this development (client perspective and intervention strategies)? The answer to this question was mitigated, but overall, cultural competency training facilitates the development of cultural self-awareness by raising issues such as awareness of biases, stereotypes, ethnocentrism, and privileges. Although training programs do not explicitly aim to develop cultural self-awareness, it appears to be an inevitable dimension that emerges spontaneously in intercultural competency training and in participants’ reflections within these programs. This confirms the centrality of cultural self-awareness in intercultural competences training.

Several studies reported that participants would like to have specific modules on cultural self-awareness in the training programs offered (Rubio Rodriguez, 2015; Smith-Augustine et al., 2014; Tomlinson-Clarke, 2000). However, this was the case only in Alraqiq et al.'s (2021) study. This absence could be explained by the fact that participants may have felt uncomfortable reflecting on their own culture, biases, and stereotypes, which could have led trainers to avoid addressing these issues (Richards & Doorenbos, 2016; Tomlinson-Clarke, 2000). In fact, Trede et al. (2013) showed that training can sometimes have the opposite effect of what is intended, resulting in the reinforcement of stereotypes. Trainers must therefore be well equipped to deal with difficult situations (discomfort, conflict). It could be hypothesized that this discomfort may also exist among trainers, making cultural self-awareness too sensitive a topic to address, and therefore possibly eliciting defensive behaviors, anger, or rejection (Debon, 2011).

Of the various intervention methods used in the studies, immersion seems to have provoked the greatest introspection in participants, leading them to question their own cultural background. Indeed, this method encourages individuals to engage directly with other cultures, to adopt new perspectives, and to confront their prejudices and stereotypes (Barden et al., 2015). This process of in-depth self-reflection can lead to a better understanding of oneself and others, facilitating the development of intercultural skills. For this reason, Merrel-James and colleagues (2019) chose this methodology for their study, believing that immersion was most likely to produce significant changes in participants’ perceptions and attitudes.

### The Lack of Integration of Cultural Self-Awareness in Training Programs and in Research

The fact that this study contains only 25 articles, of which only one focuses solely on cultural self-awareness, raises questions about why cultural self-awareness has not been more widely integrated into intercultural training. One hypothesis is that self-reflection can cause discomfort and trigger insecurities (Beaudoin-Julien et al., 2022). Trainers must therefore be equipped to manage any discomfort or potential conflicts with participants who may be reluctant to engage with the proposed content (Culhane-Pera et al., 1997; Tomlinson-Clarke, 2000). Otherwise, there can be several consequences, such as poor supervision, content that reinforces stereotypes (Cohen-Emerique, 2015), and certain topics, such as cultural self-awareness, not being addressed because they are confrontational. Ultimately, the discomfort experienced by trainers can result in defensive reactions or avoidance of certain topics, thereby contributing to the resistance observed among participants. To avoid this, people who will be providing intercultural training should undergo training themselves. However, this does not seem to be the case. Without training, trainers’ learning is often based on self-teaching or support from other trainers, which can introduce bias. Indeed, we tend to pass on what we know rather than what we do not know. Cultural self-awareness is rarely addressed, meaning that trainers may lack it. These gaps are then reproduced systematically. One possible solution would be to set up standardized, supervised training courses on intercultural competences to standardize knowledge, skills, and practices.

The same discomfort caused by self-reflection can be found in research. Just as trainers do not systematically address cultural self-awareness, researchers may also find it challenging to address this issue for similar reasons. In a 2017 action research study on intercultural workshops, White and Gratton (2017) demonstrated that certain discussions and topics can cause discomfort. The authors recommend further research into this discomfort to enable it to be addressed more effectively (White & Gratton, 2017). Furthermore, this discomfort in research can lead participants to display social desirability for fear of appearing intolerant. Several studies therefore recommend measuring social desirability when working on cultural self-awareness, to limit its influence on the results (Kemmelmeier, 2016; Müller et al., 2021; Udayar & Antonietti, 2021).

### Limitations of Included Studies

None of the included studies provided an assessment of the behavioral effects of the interventions. In fact, as shown in one of the studies, participants appeared to have remained at a cognitive level of cultural self-awareness development (Matkins et al., 2014). It would be interesting to investigate this aspect to see if training has effects on behaviors, i.e., if participants show behavior more adapted to diversity after the trainings.

The inconsistent findings also raised the question of a social desirability bias, which was never assessed in the included studies. Given the sensitivity of the topic, it is likely that participants strove to appear more open-minded than they actually were (Kemmelmeier, 2016; Muller et al., 2021; Udayar & Antonietti, 2021). This bias was particularly evident in items measuring aspects that are valued in society, such as open-mindedness (Kemmelmeier, 2016). Therefore, future research on cultural self-awareness may benefit from measuring social desirability bias.

### Study Limitations

This scoping review has some limitations. Firstly, it only looked for studies written in French and English. Secondly, there is a possibility that some may have been missed in the review due to the selection of databases, although a dozen were selected. Finally, another limitation is the use of different terms. Some authors may have used alternative terms, such as humility or cultural sensitivity, to describe the phenomenon under scrutiny, which reinforces the need for a more precise definition of cultural self-awareness, as proposed in this article. This may be due to a difference in terminology. During the research process, it became clear that different terms were used to describe cultural self-awareness. In France, for example, Cohen-Emerique used the term *decentration* (decentering), which she defined as “awareness of one's own frame of reference” (Cohen-Emerique, 2015, p. 179). However, when the French databases were searched, no studies were found, probably because the search term was precisely *conscience culturelle de soi* (cultural self-awareness). As discussed earlier, this is a limitation of this scoping review, but the different terms used for cultural self-awareness are also an avenue to explore.

### Strengths of the Study

The main strength of this study lies in its literature review. Indeed, the scoping review enables an exploratory approach to cultural self-awareness and its treatment in intercultural training. We therefore conducted an extensive literature search while adhering to the Joanna Briggs Institute's rigorous scoping review methodology (Peters et al., 2022). Additionally, sorting the articles by two evaluators with inter-rater agreement helped reduce subjective bias.

### Implications for Future Studies

For the future, it would be interesting to develop an intervention that focuses solely on the development of cultural self-awareness, defined as *the awareness of one's own prejudices, stereotypes, biases, privileges, and power relations in relation to one's cultural background(s)*, and to evaluate its effects at both cognitive and behavioral levels, considering social desirability. Taking this a step further, it would also be interesting to try to understand why cultural self-awareness is rarely addressed in training courses. Is it because trainers don’t know how to approach it, or because they feel uncomfortable doing so? There may be underlying mechanisms that prevent trainers from addressing this dimension of intercultural competences. Exploring these underlying mechanisms could enable trainers to better understand their effect and therefore further limit their impact.

Finally, the proposed new definition of cultural self-awareness emphasizes the privileges and power relations that are central to intersectionality. Intersectionality is the concept that certain aspects of identity and social status intersect to create experiences of discrimination, oppression, and privilege (Mosley et al., 2025). Therefore, it would be appropriate to incorporate intersectionality into cultural self-awareness training or, more broadly, intercultural competences training. For instance, Magalhaes et al. (2025) conducted a study among healthcare professionals and proposed training to explore how gender, ethnicity, and social class can influence women's lives and affect their health. This training consisted primarily of discussions between participants, as well as watching videos and using a discussion guide (Magalhaes et al., 2025). The results showed that the training was a transformative experience for participants, raising their awareness of certain determinants of health. In another study, Thomas et al. (2021) used scenarios with characters having multiple social identities to raise participants’ awareness. The results showed an increase in participants’ awareness and a better understanding of intersectionality and inclusion (Thomas et al., 2021).

These two studies demonstrate that developing an awareness of one's implicit biases and of the experiences of certain individuals is an integral part of intersectionality. Therefore, it would be interesting to incorporate intersectionality into intercultural competences and cultural self-awareness training courses. However, it would not be enough to simply discuss the different forms of diversity and be aware of these biases; we would also need to consider the interactions that can occur between different forms of diversity and their influence. Future research could therefore examine the link between cultural self-awareness and intersectionality.

## Conclusion

This scoping review shows that it is indeed possible to increase cultural self-awareness through training courses which promote exchange between participants or immersion. However, cultural self-awareness itself is still little studied and the tools available to adequately assess it are limited, despite a consensus on its centrality for intercultural competences (Buckley & Foldy, 2010; Cohen Emerique, 2007; Feize, 2015; Hickel, 2006). The results also indicate a need on the part of professionals to delve deeper into this dimension of intercultural competences. This study shows that there are several different methods for developing cultural self-awareness, the most effective of which seem to be those that involve immersion and interaction between participants.

## Data Availability

The authors confirm that the data supporting the findings of this study are available within the article.

## Appendices

**Table A1.** Article Extraction Table.

Article title	Author(s)	Date of publication	Sample	Participants’ gender	Participants’ backgrounds	Type of intervention	Research design	Measurement or observation tools	Main results
Cultural awareness training for dental students	Alraqiq et al.	2021	113 first-year dental students and postdoctoral dental students	56 men 57 women	65 White, 8 Black or African-American, 36 Asian, 17 Hispanic,1 American-Indian	Full-day workshop with activities	Mixed study	Interviews	First-year students: Increased ability to challenge the notion of privilege Postdoctoral students: Improved recognition of own biases
The impact of the experiential multicultural counseling program on counselor trainee cross -cultural competence	Callo	2002	17 master's students in counseling aged 23 to 47	3 men 14 women	1 Latino, 3 Asian, 3 Black, 10 Caucasian	2 days in a row 10h program with exercises	Quantitative with pre-test-post-test randomized control group design and a correlational design	Vignette videos, IIFRS et MAKASS-C	No significant difference in awareness between intervention and control groups
The influence of multicultural training on perceived multicultural counseling competencies and implicit racial prejudice	Castillo et al.	2007	84 students in counseling	17 men 67 women	65 White , 3 African-American, 5 Asian-American, 5 Latino, 6 biracial	3h class, once a week for 15 weeks	Quantitative, quasi-experimental, longitudinal approach with comparison groups	MCI and IAT	4% increase in cultural self-awareness from pre-test to post-test Higher cultural self-awareness among intervention participants than control group
An analysis of intercultural competence training for community college employees	Conway	2008	181 community college employees			6 years intercultural competence training, discussion, and theoretical information Each session lasted for 4h. Each year only 1 level of session was given	Mixed method with pre and post test measures	IDI and open questions	Perceived change in cultural self-awareness Felt that they learned something about themselves and their culture
A curriculum for multicultural education in family medicine	Culhane-Pera et al.	1997	33 medical residents			3 days in a row, workshop	Mixed study	Questionnaire and interviews	Increased knowledge and skills but not attitudes. Participants’ resistance to self-exploration, which may have affected the development of cultural self-awareness
Impact of a cultural self-analysis project: Increasing early childhood preservice teachers’ cultural competency	Fuller et al.	2006	27 students in initial training, early childhood educators Between 19 and 53 years old	27 women.	21 Caucasian, 4 Latino-Hispanic, 2 bi- or multi-racial	5 weeks of meetings	Qualitative study	Venne diagram, open questions	Participants reported greater cultural self-awareness
Promoting intercultural competence among criminal justice students using interview-based signature assignments	Havis	2019	43 students in the legal profession			3 interviews between fall 2016 and spring 2017 in the “multicultural issues in criminal justice” course	Mixed method study	AAC&U VALUE, written evaluation of participants	Participants reported greater cultural self-awareness, especially with regard to prejudice
Prejudice awareness through cultural self-knowledge development: Going beyond the contact reduces prejudice hypothesis	Hosokawa	2012	34 students in family and marital therapy, clinical psychology, social work, and gerontology			Course, interactive classes, exercises	Qualitative study	Written report	Participants realized that they didn’t know much about their own culture
Cultural sensitivity training: Description and evaluation of a workshop	Hutnik & Gregory	2008	350 nursing students			Workshop with exercises, theory, and genograms between October 2004 and October 2005	Qualitative study	Interviews	All the participants interviewed reported an improved cultural self-awareness
Cultural competence education for practicing physicians: Lessons in cultural humility, nonjudgmentall behaviors, and health beliefs elicitation	Kutob et al.	2013	90 physicians, 41 in the control group and 49 in the intervention group	31 men 59 women	60 White, 6 African-American, 15 Asian, 3 Hispanic/Latino, 3 American-Indian	“5 case-based modules, up to 9 h (dependent on how many modules were completed)”	Interactive case-based, quantitative study	CCAT	Lower cultural self-awareness in the intervention group than in the control group
Preparing science teachers for teaching in high-need schools: A comparison of two science education programs	Matkins et al.	2014	12 students with a bachelor's or master's degree in secondary science teaching	4 men 8 women		Group 1: immersion Group 2: immersion and reflection exercises on intercultural issues	Immersive, mixed study	Interviews et CRTSE (Culturally Responsive Teacher Self-Efficacy Scale)	Cultural self-awareness was important, but this reflection remained at a cognitive level
Promoting awareness of self: Cultural immersion and service-learning experiences of counselors-in-training	Merrell-James et al.	2019	11 mental health students and 1 social work student, aged 24 to 28	2 men 10 women	7 Caucasian, 4 African-American, 1 Jamaican-American	Immersion in the Caribbean during 8 days	Exploratory, qualitative, phenomenological study	Focus group	Great awareness of privilege and recurring themes: racism, attitudes, beliefs, bias
Getting to know the piece of fluff in our ears: Expanding Practitioners’ cultural self-awareness	Mirsky	2013	51 social work students	10 men 41 women		48 to 50h, meetings once a week	Interventional,qualitative study	Participants wrote between 200 and 500 words 2 months after the end of the workshop	Increased awareness of one's own immigrant experience and the immigrant history of one's family Awareness of one's own life transitions
Developing a global curriculum in a school of nursing	Parcells & Baernholdt	2014	15 nursing school students			Workshops, one that lasted 4h in January 2013, another that lasted 5h 30 in September 2013	Formative and qualitative study	Student evaluation	Students reported that they saw cultural self-awareness as the first step toward more culturally sensitive teaching, and they appreciated the part of the workshop on stereotypes and self-awareness
Transformative learning for counselor trainees: Implementation of the multicultural immersion experience model	Prosek & Michel	2016	13 counselor trainees on a master’s degree course	1 man 12 women	10 Euro-American, 1 African-American, 1 Hispanic, 1 multiracial	13-week course then the participants spent 10 days in immersion in Ireland	Short-term immersive, qualitative study	Group discussions	Students reported recognizing their own ethnocentric viewpoints, reflecting on their cultural origins, stepping out of their comfort zones, and seeming to increase their cultural self-awareness
Intercultural competency development of health professions students during study abroad in India	Richards & Doorenbos	2016	18 students in health-related professions	1 man 17 women	10 White, 8 from other ethnic groups	3-week course in India (immersion) with a preparatory meeting 3 months before departure	Immersive, mixed study	IES, ISS, and logbooks	No difference pre and post for IES and ISS Logbooks: Level 2 cultural self-awareness: could identify own biases, but had a preference for the rules of their own culture
Examining the process of the development of multicultural counselling competencies in therapist-trainees	Rubio Rodriguez	2015	14 therapist-trainees, doctoral level in clinical psychology	1 man 13 women	11 White, 3 East-Asian	Use a critical incident log between encounters with refugee users	Longitudinal, qualitative study	Analyzed responses to critical incident log questions	Felt the need to develop their cultural self-awareness Learned about and questioned their own culture
Service providers’ cultural self-awareness and responsible use of racial power when working with ethnic minority victims/survivors of child sexual abuse: Results from a program evaluation study in Australia	Sawrikar	2020	T1: 122 T2: 44 mental health practitioners (psychologists, psychiatrists, social workers, counselors)	21 men 101 women	63 White/Caucasian, 59 other ethnic origin	Classes that last half a day	Mixed, longitudinal study with data triangulation	Questionnaire included open-ended questions	Quantitative: slight increase in cultural self-awareness among ethnic minority service providers Qualitative: allowed reflection on the issue of white privilege and one's own interpretations
International immersion in Belize: Fostering counseling students’ cultural self-awareness	Smith-Augustine et al.	2014	5 master's level students in counseling between 20 and 25 years old	5 women	5 African-American	3 weeks immersion to Belize	Immersive, qualitative study	Logbook	Participants want to know more about their own cultural heritage Recognized that they were less culturally sensitive than they thought, and that they had biases, stereotypes, and prejudices about others
Building teacher interculturality: Student partnerships in university classrooms	Smolcic & Arends	2017	16 pre-service teachers, between 19 and 22 years old	2 men 14 women	15 White/Caucasian, 1 Asian	Autobiography and encounter with international students weekly for at least 1h during 8 weeks	Collaborative, qualitative study	Blog maintained by participants + cultural analysis paper + final reflective paper	Working with international people stimulated introspection and cultural self-awareness Realization that they knew less about their own culture than they thought, and that they were less culturally competent than they thought Awareness of stereotypes and representations, and questions about the concept of power in society
The impact of a cultural competency course on the racial identity of MSWs	Spears	2004	33 students in social work	7 men 26 women	28 White/Euro-American, 1 Latino-Hispanic, 4 biracial	15 weeks of classes	Quasi-experimental mixed study	Narrative inquiry	Developed a critical eye for their own biases
Assessing outcomes in a multicultural training course: A qualitative study	Tomlinson-Clarke	2000	17 graduate students in counseling psychology		6 White, 5 Black / African American, 4 Asian, 2 Latino	Classes once a week for 2h 40 for 15 weeks	Interventional, longitudinal, qualitative study	Written course evaluation and interviews	One participant reported that he became aware of how unaware he was of his own biases Most participants found it difficult to self-analyze their own biases
Developing measurable cultural competence and cultural humility: An application of the cultural formulation	Tormala et al.	2018	30 2nd-year students in clinical psychology, doctoral level			Clinical vignettes and written feedback	Comparative, qualitative study	Answers to vignettes	50% of participants showed an increase in cultural self-awareness between the two vignettes
Developing cultural awareness and sensitivity: An experiential approach	Wesselkamper	1983	65 graduate social work students	14 men 51 women	51 White, 14 minorities	Class during a semester group discussion for 5 weeks and presentation of the discussion	Mixed, exploratory, and experiential study	Recording exchanges	Discussions on stereotypes, prejudice and ethnocentrism
Self-awareness and cultural identity as an effort to reduce bias in medicine	White et al.	2018	18 first-, second-, and fourth-year medical student participants			14, 2h Interactive classes with videos and readings, as well as genograms	Qualitative study with a participatory and reflective approach	Participants’ writings	The participants realized that they had biases and that they also had blind spots
